# Design and Test of a Hybrid Foot Force Sensing and GPS System for Richer User Mobility Activity Recognition

**DOI:** 10.3390/s131114918

**Published:** 2013-11-01

**Authors:** Zelun Zhang, Stefan Poslad

**Affiliations:** School of Electronic Engineering and Computer Science, Queen Mary University of London, Mile End Road, London E1 4NS, UK; E-Mail: stefan.poslad@qmul.ac.uk

**Keywords:** mobility profiling, activity recognition, foot force sensor, GPS, accelerometer

## Abstract

Wearable and accompanied sensors and devices are increasingly being used for user activity recognition. However, typical GPS-based and accelerometer-based (ACC) methods face three main challenges: a low recognition accuracy; a coarse recognition capability, *i.e.*, they cannot recognise both human posture (during travelling) and transportation mode simultaneously, and a relatively high computational complexity. Here, a new GPS and Foot-Force (GPS + FF) sensor method is proposed to overcome these challenges that leverages a set of wearable FF sensors in combination with GPS, e.g., in a mobile phone. User mobility activities that can be recognised include both daily user postures and common transportation modes: sitting, standing, walking, cycling, bus passenger, car passenger (including private cars and taxis) and car driver. The novelty of this work is that our approach provides a more comprehensive recognition capability in terms of reliably recognising both human posture and transportation mode simultaneously during travel. In addition, by comparing the new GPS + FF method with both an ACC method (62% accuracy) and a GPS + ACC based method (70% accuracy) as baseline methods, it obtains a higher accuracy (95%) with less computational complexity, when tested on a dataset obtained from ten individuals.

## 1. Introduction

User mobility or activity is an important type of user context that can be used as a knowledge source to better tailor and adapt a raft of rich applications to users' needs in different mobility-related situations. The increasing use of wearable and accompanied device body sensors networked as body area networks adds a new type of sensor data to help promote an Internet of Things. These sensors can also act as an enabler for the hidden computer part of Weiser's ubiquitous computing vision to increase the implicit human computer interaction (iHCI) with systems and services through reducing users' cognitive load, distractions and informational overload when users respond to the myriad of intelligent devices and sensors in their immediate environment [1]. A wider range recognition of user activities could facilitate many useful applications [2]. These include: Health and Physical Activity Monitoring [3,4]; Individual Environmental Impact Monitoring [5,6]; Crowd Mobility Awareness [7,8]; Mobility-aware Service Adaptation [9].

### 1.1. Profiling Human Mobility

Mobility may be classified in different ways across a broad range of users' mobile activities and transportation modes to enable the above applications. Locations determined on-route can be used to help differentiate transport modes. However, the use of simple fixed location heuristics to classify modes may be error-prone, e.g., taxis may travel on bus routes because they are less congested.

Velocity or acceleration (derived from location changes with time) can also be used to differentiate different types of mobility as the average movement velocity for free-flowing people and vehicles vary across transportation modes, e.g., velocity increases from walking, to cycling to taking a bus. However, these modes' velocities and accelerations can vary and overlap. The speed of movement between motorised and non-motorised individuals varies based upon ability, the propensity for speed and due to environmental conditions, e.g., for a bus that is stuck in congestion, cycling or even walking may be quicker. Road vehicle speed is limited by law, but this varies. Hence, use of a simple threshold for speed, to differentiate between motorised and non-motorised mobility, or differentiate different sub-types of motorised modes (use of a car or taxi, bus) or differentiate sub-types of non-motorised modes (standing, walking, or cycling), is quite complex.

Whole body posture, *i.e.*, standing *versus* sitting, varies between different transport modes, e.g., people sit on a bike, car or taxi but stand while walking but people may remain standing, or walk to get to seat, on a bus or train but not in a car, taxi or bike. Thus, a simple classification of whole body posture alone, if it could be detected, cannot differentiate consistently the use of different sub-classes of motorised transport. In addition, it may be useful to differentiate both posture and transportation mode in order to be able to differentiate users walking unaided *versus* travelling in a moving public transport vehicle, in which they happen to be walking. For some types of on-route transport information service, it is useful to differentiate a driver *versus* a passenger. For example, bus drivers may require route navigation information but bus passengers are more concerned with knowing which bus stop is the closest stop to a destination and where to get off the bus, rather than seeing the whole bus route. It may also be less safe to distract a road vehicle driver with an incoming or outgoing phone call than to distract a passenger.

### 1.2. Sensing Human Mobility

The earliest human mobility monitoring systems used sensors fixed into the environment, such as foot-force plates, that were often combined with on-body tags rather than sensors whose movement could then be visually captured using video cameras and then analysed to detect the tag movement [10–12]. Fixed environment tags or sensors can provide accurate, calibrated, measurements of human motion, however their chief disadvantage is that these cannot be used for pervasive monitoring of people during daily life.

Key technology enablers for pervasive user mobility context awareness are firstly, inertial sensors, such as an accelerometer, gyroscope or compass, manufactured as a Micro Electro-Mechanical System (MEMS). Research has shown that there is a good agreement between on-body motion sensors and fixed environment motion sensor measurements [13,14]. The accelerometer is the most popular inertial sensor used for activity detection, while other inertial sensors, such as gyroscope and compass, are mainly used as assistive sensors due to their limitations in detecting user activities alone [15]. In addition, the accuracy of accelerometer-based method is also affected by different body motion such as bending, swaying and twitching [2]. The accelerometer may not sometimes recognise the user or human posture during travel, as the acceleration patterns from a user's motion and a vehicle's vibration can overlap [2].

Second, sensors that are wearable can be utilised for activity monitoring [16,17]. There are well-defined foot movements and foot forces generated when walking or pedalling a cycle that can make these types of motion relatively easy to sense. More recently, commercial wearable sensors have become available to profile user activities by analysing data from wearable sensors, at fixed body positions, on mobile devices. An example commercially available wearable sensor system is the Nike + iPod system. This mounts a single sensor that can be used as a pedometer inside one shoe in a pair of Nike running shoes connected to an iPod device that acts as a data hub. This can be used to profile users jogging [18]. This senses one specific type of user mobility, *i.e.*, walking (or jogging or running), *via* the foot pressure surges, as someone repeatedly steps on the ground. As only one sensor is used for the whole of one foot, the system does not monitor the full value of ground reaction force generated from one foot. This limits the system from detecting fine-grained human postures, e.g., differentiating between standing and sitting. In addition, by only sensing the movement in one foot rather than in both feet, it cannot differentiate other mobility activities that involve both feet, e.g., cycling and driving a car. These limitations may also introduce more errors in differentiating between a body rocking and swaying *versus* stepping.

Single wearable sensor based methods, whilst to some extent achieving some useful mobility recognition results, tend to suffer some common limitations such as low accuracy, narrow range and a coarse mobility recognition capability [16,19,20]. In contrast, multi-sensor based methods that combine two or more sensors normally outperform the single-sensor based methods in terms of a higher accuracy but they also require more resources, e.g., have a higher computation, higher cost, and can be harder to maintain [21,22]. Despite the added deployment challenges, multi-sensor based methods and hybrid sensor methods that combine wearable sensors and mobile or accompanied device sensors, have received increasing attention [18,23].

In contrast to a single wearable sensor used as a pedometer, multi-sensor types of wearable foot force sensor system can be used to capture richer and more finely grained user foot force variations caused by different human postures, e.g., standing and sitting and activities, e.g., cycling and driving in real time [14]. However, the use of the foot force sensors to support richer mobility activities recognition also faces significant challenges. Different mobility activities may exhibit similar foot force patterns, which can be hard to differentiate, e.g., car passengers and seated bus passengers sometimes generate quite similar foot force patterns. This can be addressed through the joint inference with other sensor types, e.g., GPS. The variability in where sensors are placed can produce different sensor measurements. This can be addressed, when it is feasible, by fixing the sensor position, e.g., using a standard shoe inset. For the same type of user activity, user movement may vary. This makes it more difficult to compare sensor readings across different subjects, because the foot force signal noise due to small body movements, such as swaying, varies. However, many mobility activities involve a regular shift of pressure between the left and right foot such as walking and cycling, the accuracy of detecting and classifying these activities can be improved if a method can monitor this pressure shift and use this to classify these activities.

Third, although inertial sensors can be worn as individual macro sensors by themselves, they can also be integrated into more complex mobile devices such as smart phones that accompany a user. The different positions for accompanied, *i.e.*, mobile phone, sensors, e.g., held in the hand, in clothing or in a bag, rather than being worn in a fixed position or even left on another stationary or moving object where its motion maybe unrelated to body motion, can produce different sensor measurements. Hence one needs to be able to differentiate different sensor measurement values due to different positions or configurations and those due to different movements.

The rising memory and processing power of the smart phone enables it to act as a local data processing and information storage hub or as a relay for data from body area networks of wearable sensors [24,25]. In addition to the integrated inertial sensors, mobile phones have integrated transceiver type positions sensors such as GPS, WiFi and GSM that use in-network measurements of signal time arrival and signal strength to determine user spatial contexts such as location and speed [26,27]. These are able to support a range of user context aware services during everyday activities [28,29]. Some of these services are participatory, in which the user is involved in significant decision stages of the sensing systems sensors, but the majority are opportunistic, in which decisions about sensing are taken by the system on behalf of users and in which users are not directly involved [30].

If on-demand access to in-network processing services via a mobile device is available then the analysis of the sensor data can be analysed online and reported back to the mobile devices. There are pros and cons to performing the sensor data analysis on body (on the mobile device) *versus* performing the sensor data analysis off-body or remotely. The benefits of performing local analysis, on-body, on the mobile device are first that the data is not shared with remote services and can be kept private. Second, it does not require a wireless on demand data connection to a remote server that can be subject to intermittent interference and a subsequent lack of service access. Third, local data analysis can also lead to better near real-time data classification and travel service information adaptation, providing the computation is light enough to be performed on mobile devices.

### 1.3. Research Aims, Challenges and Contributions

Our primary research aim is to assess how well a combination of mobile phone GPS and wearable foot force (FF) sensors (GPS + FF) recognises different user daily mobility activities, compared to a state of art inertial sensor use, e.g., accelerometer (ACC) [20] and a GPS+ACC combination. We focus on local data analysis, on device because of the benefits given in Section 1.2. Improvements to the mobile profiling accuracy are investigated in terms of how to get a more fine grained recognition and how to diminish the computational cost of mobility activity recognition. The proposed strategies are first validated on a dataset composed of samples of 10 individuals and then through comparing our GPS + FF sensor approach with both an ACC based method and a GPS + ACC based one. The key challenge here is how to design the system and experiments in a way to take into account the variability effects in different datasets (collected multiple times for generating classification results with different sensor based methods) arising from the challenges given above.

The specific contributions of this paper are as follows: First, a thorough survey of methods in both human posture and transportation mode recognition has been conducted; Second, both an accelerometer-based method (identified as the best practice method in the survey) and a GPS + ACC based method have been reproduced to provide baseline methods for the evaluation of a new (GPS + FF) method; Third, a new method for mobility context awareness that leverages a set of foot force sensors and mobile phone GPS has been designed and implemented; Fourth, the GPS + FF method has been experimentally evaluated in 10 test subjects against both an ACC based method and a GPS + ACC based method to validate its benefits.

## 2. Related Work

Currently, the most popular types of sensors used for user mobility determination are inertial sensors (mainly accelerometer) and GPS. Typically, these sensors are embedded into widely used smart phones. Hence, smart phones are commonly used by researchers as user mobility sensing devices. Hence, the use of accelerometer and GPS is a focus in this survey. The second main focus in this survey is on the use of FF sensors, including hybrid FF sensor techniques. The critical analysis of related work is partitioned according to different sensor configurations–single sensor based and hybrid sensor based.

### 2.1. Single Sensor Configuration

#### 2.1.1. Accelerometer

Accelerometer measurements are a typical way to recognise types of user activity. Mizzel *et al.* [31] showed that the accelerometer signal can produce a good estimate of the vertical and horizontal acceleration components. The vector holds an estimation of the magnitude of the dynamic acceleration of the human host that carries the sensor device. Different user activities, such as walking and cycling, may generate different acceleration patterns that can be differentiated. Ravi *et al.* [19] have found that several user activities can be recognised with a reasonable accuracy by wearing a single triaxial accelerometer near the pelvic region. Bao *et al.* [32] used five biaxial accelerometers worn around different parts of the body to recognise different user activities. Their results show that the thigh and wrist sensor placements can recognise everyday activities with an overall accuracy rate of 84%. In [33], Juha *et al.* utilised a wireless motion band attached to a user's ankle to sense the acceleration generated by the ankle during different activities. This work has successfully differentiated different user daily activities such as walking, running and cycling through using a binary decision tree feature classification method. A personalised classification method also increases the accuracy of detection. Similar work has also been done by Myong-Woo in [34] and by Brezmes in [35].

Accelerometer-based methods can achieve an increased accuracy when people carry their smart phones in a fixed place. However, people normally tend to carry their mobile phones more freely, such as near the waist, in a front pocket, in a knee-high pocket, by hand and so on. The use of the accelerometer for classification is limited because different on-body placements of the device will greatly change the nature of the motion signal and cause noises, which finally leads to a low accuracy of specific placement trained classifiers for free use. Wang *et al.* [20] have also considered this issue and attempted to differentiate user activities without any placement restrictions for accelerometers. They used a smart phone embedded accelerometer to recognise six kinds of transportation mode, but the accuracy is relatively low at 62% on average.

#### 2.1.2. GPS

GPS, as a global-wide positioning system, has already been integrated into mobile phones. The potential usability of GPS in profiling user daily outdoor activities has been widely presented, such as in [16,36]. Lin Liao *et al.* [16] have developed a probabilistic temporal model that can extract high-level human activities from a sequence of GPS readings. Two main types of transportation mode (human powered and motorised) are inferred based on the Conditional Random Fields model. Though they achieved over 80% percentage in accuracy, the range of the transportation mode recognised is coarse—it can only detect two main types of transportation mode, human powered and motorised. In addition, this method cannot differentiate between different transportation modes with similar speed characteristics, e.g. a slow travelling bus during traffic congestion can be miss-classified as cycling.

In contrast to [16], Zheng *et al.* used a supervised learning based approach to infer more kinds of transportation modes from the raw GPS data in [36]. They proposed a change point (between different transportation modes) based upon a segmentation method. The results show that the change point based segmentation achieved a better accuracy compared with uniform-duration based segmentation and uniform-length based segmentation. However, GPS information alone cannot detect change point precisely, since on many occasions, a person could take a taxi immediately after he or she gets off a bus and this very short changing segment between these two transportation modes can be hard to detect using GPS alone.

Existing GPS research exposes an inherent limitation of the single GPS-based method. GPS information alone is too coarse to enable human posture and more fine-grained transportation mode recognition with a good accuracy. For example, GPS performs poorly for the recognition of different transportation modes with similar speeds such as with fast walking, cycling and slow motorized travel. GPS based method can only be used to recognise transportation modes with marked speed differences and cannot detect stationary postures and indoor activities.

#### 2.1.3. Foot Force Sensors

It is well known that different user activities may generate different ground reaction forces [14]. Hence, suitable lightweight sensors are needed to instrument the body to provide user data pertaining to user activities in daily life environments. Nowadays, there is a range of sensor based human posture and activity detection research, such as [13,14,22,33,37–40]. In this research, data from normal behaviour can be gathered from wearable sensors. However, most of the work focuses on indoor usage and does not examine its potential usefulness in recognising user mobility activities in daily life, the awareness of which is considered as an important part of the vision of ubiquitous computing [41].

Veltink *et al.* [42] measure the ground reaction forces and centres of pressure (CoP) using two six-degrees-of-freedom movement sensors under each shoe. By comparing their measurements with the ground reaction force measured by a fixed environment foot force plate, this work illustrates the potential usefulness and feasibility of using portable foot force sensors in detecting user activities. This work also shows that ambulatory measurement of user movement is feasible through capturing the force generated from both the heel and forefoot for each foot. However, this work only measured the foot ground reaction force when walking. Other mobility activities were excluded from the research. There are also other limitations of this work. The pair of experimental shoes was instrumented with 6-axis force and moment sensors, which is too cumbersome (15.7 mm in thickness) to be worn daily. Another limitation of this work is that only one test subject has been included. Similar work has also been done by Tao [43] and by Zhang [44].

Zhang *et al.* [14] assessed human activities such as walking, jogging and running by using a small, non-intrusive insole pressure measurement device. This can be used to estimate the speed of walking and can be used in everyday life. They studied 40 subjects and achieved a fairly high accuracy of human activity recognition (95%). One of obvious drawbacks of this work is that only user activities involving walking and running are considered. Important daily human postures during travelling such as standing, sitting are not included. Another limitation of this work is that other user mobility activities involving common daily transportation modes, e.g., cycling, motorised modes, have not been studied. Although there are obvious limitations of this work, nevertheless the potential usefulness of using foot force sensors to recognise daily user mobility activities has been illustrated.

The foot force sensing systems mentioned above are wire-based. This means the force sensors are connected for power and the monitoring data are transmitted via wires to a receiver. In order to extend the foot force sensors based methods to a ubiquitous use, a more non-intrusive wireless way is required. Tracie *et al.* [13] designed and implemented a Wireless In-shoe Force System (WIFS) to acquire, process and transmit FF sensor information. This pilot study showed the feasibility for using a portable foot force monitoring system in a variety of locations rather than in a laboratory setting. In addition, this work also proved that using a limited number of FF sensors, 4 on each foot, as long as they are properly arranged under the supporting bones of each foot, enables accurate foot monitoring information to be obtained, when compared with force plated monitoring as the ground truth. However, the key limitation of this work is that only mobility activities such as walking and standing are considered. Similar wireless pressure-sensitive foot insoles have also been done by Macro in [45] as part of their sensor system.

In summary, foot force sensors can be used to recognise foot related activities at a fairly high accuracy using a limited number of sensors. Existing single FF sensor based methods' usefulness in recognising daily user mobility activities are limited because many mobility activities cannot be recognised by FF sensor alone, e.g., driving a car, because different mobility activities may exhibit similar foot force patterns. Based upon this, a hybrid-FF based method is required to provide extra spatial contexts information for better mobility activity recognition.

### 2.2. Hybrid Sensor Configuration

Yang *et al.* in [39] make use of three body-worn sensor boards to detect abnormal human activities. Abnormal activities such as slipping on the ground, falling down forward and falling down backward can be detected with over 90% accuracy. Other activities such as walking and running are also included. The limitations of this work are as follows. First, for each sensor board, there are five different types of sensors included such as light, temperature, microphone, 2D-accelerometer and two-axis magnetometer. This means a user carries at least 15 (3 × 5) different sensors. This is an intrusive device that affects and restricts the normal behaviour of the user. Second, no stationary posture, such as sitting and standing or outdoor transportation modes were studied.

In [46], Chon and his colleagues presented a smartphone-based context location aware system that fuses accelerometer, Wi-Fi and GPS to track and to automatically identify points of interest with room-level accuracy. The inbuilt smart phone accelerometer sensor and Points of Interest (POI) are used to capture and represent user activities. This has the benefit of not requiring any specialised instrumented environment and not requiring extra sensors to be worn on the human body. However, there are limitations: the use of GPS combined with accelerometer for outdoor activities tracking. These are not capable of detecting richer user mobility contexts such as when a user is standing on a moving bus. In addition, other relevant mobility activities such as walking and cycling are ignored by the author.

Varkey *et al.* in [47] utilised a set of support vector machines (SVM) to recognise user human motion in real time using a wearable wireless sensor-based system that contains an accelerometer and gyroscope. This can recognise six different activities-walking, standing, writing, smoking, jacks and jogging. When tested on three different subjects, the accuracy of the proposed system in detecting the required activities is around 84%. A key limitation of this work is that two devices are required to be placed on two fixed positions, on the right arm wrist and on the right foot, in order to acquire the linear acceleration and angular rate. In daily living, people tend to carry their mobile devices more freely. A more flexible method with no placement restrictions is required. Another limitation is that although several mobility activities are detected, other useful mobility activities such as transportation modes are not considered.

Reddy *et al.* [23] proposed the use of both accelerometer and GPS to recognise different transportation modes. Features are extracted from a series of acceleration magnitude readings, which represent the value of the three axis acceleration vector magnitude. This work can effectively discriminate human powered transportation mode such as walking and cycling. However, it is unable to provide a more fine-grained recognition capability such as sub-differentiating motorised mode into taking a car, driving or taking a bus. And this method cannot detect both human posture and transportation mode simultaneously, which is important in enabling smart services. Moreover, this work utilised a complex two stage classifier (Decision Tree + Discrete Hidden Markov Model), which is quite computational expensive to use in mobile devices.

Minnen *et al.* [22] utilise three microphones, two accelerometers and a wearable computer to recognize different user activities. By mounting microphones on the chest, elbow and right hand respectively, by comparing the sound intensity of these three microphones, this method can be used to automatically index the captured journals of a person's life. Further, by attaching two 3D accelerometers on each wrist, the motion pattern of both hands can be captured. A comparison of the acceleration generated between the left hand and right hand is used to infer daily activities such as hammering and sawing. The limitations of this work, first, are that the activities that can be recognised by this system have to be sonant; otherwise the use of the microphone is useless. Second, other mobility activities related to the motion of legs and foot, such as walking, standing and cycling, are not considered. Similar work has also been done by Takuya in [48].

Weijun *et al.* [17] used three accelerometers, three gyroscopes and five tri-axial force sensors to recognise user activities. By mounting three pairs of accelerometers and gyroscopes on three fixed positions (foot, calf and thigh) in combination with a set of foot force sensors, the system achieved a very detailed ambulatory gait analysis capability. By dividing a normal gait cycle into four gait phases and four swing periods, it can provide useful information for multiple health-related applications. This work proved that by combining with other sensor types, foot force sensors are extendible to provide more fine-grained activity recognition capability. However, the scope of this work is narrow (only walking is considered) and excludes a wide range of mobility activity recognition such as transportation mode recognition.

In summary, current hybrid-sensor-based methods achieve a higher accuracy, compared with single sensor based methods. However, they still tend to lack of support for wider range mobility activity recognition in daily living environment.

### 2.3. Classification of Analysed Systems

Table 1 classifies user mobility with respect to multiple dimensions, the number and types of sensors, sensor position, the types of user mobility, the types of features extracted, the classifiers used, and the classification accuracy. The 6th dimension, the classification accuracy, is affected by the first five dimensions and these all vary across the related work. The average accuracy for current user mobility recognition is comparatively low, about 70%, *i.e.*, only a little over two thirds of trips are recognised correctly. Moreover, this error maybe amplified for the daily activities that are logged, *i.e.*, with an overall accuracy at level of 70%, around 3 h of data may be misclassified given that 10 h of activities are logged typically per day. This offers a good opportunity to increase its accuracy.

There is no single method that can sub-classify stationary postures into sitting and standing. Although, the related work seems to perform well in differentiating stationary and dynamic postures, the recognition of more fine grained dynamic postures, *i.e.*, walking and cycling and fine grained stationary postures still need to be improved. The majority of the related work does not support sub-differentiating motorised transportation modes. However, for potential applications such as user mobility profiling and individual environmental impact monitoring, the motorised transportation mode needs to be sub classified into more specific types, *i.e.*, car-passenger, bus-passenger and car-driver. This is because these different sub types of motorised mode may have quite different characteristics in terms of user needs and hazard exposure level. *i.e.*, generally speaking, travelling by bus is more eco-friendly than travelling by private car (assuming the car is not carrying more passengers than the bus and not using a more eco-friendly type of fuel).

Most of the surveyed systems have restrictions depending on how users should carry their (accompanied) mobile devices except [20]. Work [20] also recognises more activities and has more sub-classes of motorised transportation mode (bus passenger, car passenger) compared to other work, which better fits one of the aims in this paper–a wider range of mobility activity recognition. In addition, [20] only used a single stage classifier which fits one of our aims in this paper, a low computational complexity (see Section 3.1). Though work [25] which uses both GPS and accelerometer achieved the best accuracy, they utilised a two stage classification method, *i.e.*, DTt + HMM. Clearly, the accuracy of mobility activity detection maybe higher if one utilises multistage classifications or more complex models. However, the objective of our work is to determine the value add of the new sensor combination of GPS + FF compared with the use of accelerometer-based methods for daily mobility activity recognition. Hence, the accelerometer-based, single-stage classifier, method used in [20] is chosen as a baseline to evaluate the FF method in terms of recognising both human posture and transportation mode. As our new method (see Sections 1.2, 3.2) also uses GPS as a assistive sensor to measure speed, so the work [20] is also extended to form a GPS + ACC based method by adding the GPS as a assistive sensor as well. The recognition results from this reproduced GPS+ACC method will be used to validate the GPS + FF method. The other existing GPS + ACC based methods, such as [21,23], are not considered because they all employed advanced classification models, e.g., the two stage classification model used in [25]. In addition, we believe using the same GPS speed related features in both the GPS + ACC method and our GPS + FF method, we can achieve a fair and useful comparison between FF and ACC for user activity recognition.

One of the novelties of the GPS + FF method is that our aim is to design it to support a wider range of user mobility activities. For example, the aim is not only to recognise whether a person is taking a bus, but also to provide more information about whether that person is standing or sitting on a moving bus. This is because for the same kind of transportation mode, different human postures (during travelling) may require different kinds of transportation information and adaptation. It is also noted there is no single sensor method that can recognise both human posture and transportation mode simultaneously. Using a scenario when a user is standing on a moving bus as an example, current GPS methods appear too coarse-grained to recognise human posture during travel. The acceleration signal from both user motion and vehicle vibration may overlap with each other [2]. This makes it difficult to recognise both human posture and transportation mode simultaneously at a high accuracy.

Typical sensor based methods using accelerometers or/and GPS face some key limitations in recognising mobility activities. For accelerometer-based methods, the key limitations are:

➢ *Varying on-body placements*: People normally tend to carry smart phones more freely (waist, front pocket, knee-high pocket, hand and so on) in their daily living environment, which greatly changes the nature of the motion signal [2]. For instance, walking, running and cycling tend to exhibit similar accelerometer characteristics in certain areas of the body.
➢ *User variability*: As the accelerometer-based method requires the sensor to be carried along with users, the sensed acceleration signal changes according to the natural body motion, which may vary from user to user. For example, typical nature body motions (such as bending, swaying and twitching) sometimes may be dominant and affect the recognition accuracy of the accelerometer-based method.
➢ *Overlapping sensor signal*: Typical accelerometer-based methods can recognise human posture or transportation mode. However, accelerometer-based methods may not be able to recognise both human posture and transportation mode at the same time. This is because the acceleration signals from both user motion and vehicle vibration (during travelling) may overlap with each other [2]. This overlap highly affects the recognition accuracy for either human postures or transportation modes.

For the single GPS-based method, the common limitations are:

➢ *Loss of signal:* there is no GPS signal indoors, underground, under bridges or tunnels, between narrow buildings and inside some moving vehicles when seated as a passenger.
➢ *Remote Server:* Many existing GPS-based methods rely on remote servers to support mobility activity recognition. For example, the use of GIS, Geographical Information System, to plot user locations and moving trajectories on maps to assist transportation mode recognition [50,52]. However, all remote server based methods tend to exhaust the mobile device power level, as they need frequent data transmission. In addition, this kind of continuous user location plotting on backend servers is also at risk of privacy infringement [16,50].
➢ *Coarse grained recognition:* The single GPS-based method is not capable of providing fine-grained human posture recognition, *i.e.*, GPS-based methods cannot sub-differentiate stationary posture into standing and sitting. Moreover, the GPS speed reading is also too coarse to differentiate user mobility activities with similar speeds, such as running quickly, cycling or slow motorised travelling [16].

## 3. Method

### 3.1. Design Issues

Before describing the design of the new mobility profiling system, the design requirements in order to develop a mobile device daily activity recognition system are discussed. Based on the surveyed research, the following requirements are proposed for the user daily mobility activity recognition system:

➢ *Wider and Fine-Grained Range Mobility Recognition Capability*: In order to better understand user contexts for interacting with services in daily life, richer mobility activity recognition is needed in terms of both a fine-grained recognition capability and the ability to recognise both human postures and transportation modes, possibly simultaneously. A fine-grained recognition capability is required, because people in different mobility contexts may have different requirements. Consider the following scenario: when detecting that a user is driving a car, a mobile phone may automatically divert a call in order to ensure the user's safety on the road, while this is not necessary when detecting that the user is a passenger in a car. So the traditional travelling-by-car mode needs to be sub-differentiated into driving or passenger. It is also found that given the same transportation mode, different human postures may lead to different user requirements for transportation information adaptation. For example, when detecting that a user is standing, or walking to a seat, rather than sitting in a fast moving bus, map views and controls may be adjusted to highlight travel information more than normal, e.g., display larger labels and controls. In order to better serve this purpose, the system should be able to recognise both human posture and transportation mode at the same time and also be able to sub-classify motorised transportation mode into bus-passenger, car-passenger and car-driver.
➢ *Lightweight Local Mobility Data Computation*: The benefits of performing local data analysis, on-body, have already been given (Section 1). Current mobile devices, whilst increasing in computing power and functionality, their processing capability is still limited compared to personal computers, servers and embedded systems, with specialised hardware such as digital signal processors. In addition, mobile devices cannot dedicate their full computing resources to auxiliary applications given its primary roles are interaction and communication. Based on opportunistic, changing, local mobility activity, continuous computation is needed, without exceeding the local computational resources [40].
➢ *Sensor Error Tolerance*: A system should be able to tolerate sensor errors arising in a typical daily living environment, e.g., occasional GPS data inaccuracy and interruption. Moreover, it will be more computational efficient if a system can tolerate these occasional sensor errors, rather than continuously requiring additional data pre-possessing.
➢ *High Mobility Classification Accuracy*: According to the survey (summarised in Table 1), the average accuracy of current transportation mode recognition methods is approximately 75%. This accuracy statistically means one in every four samples will be misclassified. This offers a good opportunity to increase its accuracy. In order to satisfy the potential applications (mentioned in the introduction), the accuracy of the recognition method needs to be improved at a higher level [53].
➢ *No On-Body Placement Restrictions for Accompanied Mobile Devices*: People tend to carry their mobile phone in variable places and orientations. For some sensor signals, e.g., from accelerometers, the signal depends heavily on the sensor body position and orientation where other (accompanied) sensors, e.g., mobile phone GPS signal, is not dependent on sensor body position. A pervasive system should support such flexibility in terms of the position and orientation of the mobile phone [53].
➢ *Reduced Training to Classify Individuals*: a generalized method can be used with new users without requiring much individual user training [54]. Most existing systems for mobility activity recognition did not employ a generalised method. In these cases, they require a training phase for new users in order to conduct individual-specific training to personalise the system so as to use it with a high degree of accuracy [55]. A mobility activity recognition system should require minimal individual training.

### 3.2. Rationale for Choosing GPS + FF

According to the survey (Table 1), no single ACC, GPS or FF sensor method can meet our system requirements (Section 3.1). With respect to transportation mode, the GPS speed alone is not capable of sub-differentiating motorised transportation mode, since in many cases, e.g., fast walking, cycling and slow motorised travelling, the speed contexts are quite similar. GPS alone is not accurate enough for fine-grained transportation mode recognition. With regard to human posture recognition, there are well known foot force variations between different stationary postures, such as sitting and standing. Foot force patterns are also different human powered transport modes such as between cycling and walking.

Hence, a hybrid method is proposed that leverage both mobile phone GPS and a set of foot force sensors. The rationale for combining these two types of sensors is because of the different, and in some cases complementary, variations in sensor data in different mobility activities. Activities with a similar GPS speed pattern have different foot force patterns and vice versa (Table 2).

### 3.3. System Overview

To the best of our knowledge, the use of GPS in combination with foot force sensors to improve mobility activity recognition in a pervasive setting has not been proposed or examined in depth to date. In order to provide richer mobility contexts in terms of recognising both human postures (during travelling) and transportation mode, in the GPS + FF method, the human posture will be inferred from the foot force sensors' data, while the transportation mode is jointly inferred from both foot force sensors and GPS data. This is because based on our survey and analysis, the use of foot force sensors alone are capable of recognising various foot related human postures at a fairly high accuracy, while the additional spatial context of GPS position changes, is only required for recognising fine-grained transportation modes with similar foot force patterns (see Section 3.2). The scope includes different human postures and mobility (sitting, standing, walking and cycling) and different human-powered and motorised transportation modes (walking, cycling, bus-passenger, car-passenger and car-driver) that are most often used during daily travel. Standing and sitting postures include both the scenario of standing/sitting stationary only (e.g., at bus stops) and the scenario of standing/sitting in a moving vehicle (e.g., in a bus). Walking also includes both jogging and running during travel, e.g., people may run to a bus-stop to try to catch up to a leaving bus. Walking and cycling may be considered by some researchers as both human postures and transportation modes [53].

Therefore, the following system architecture is proposed to examine how well foot force sensors in combination with mobile phone GPS can recognise both human postures and transportation modes, compared with other typical methods.

**Figure 1.**
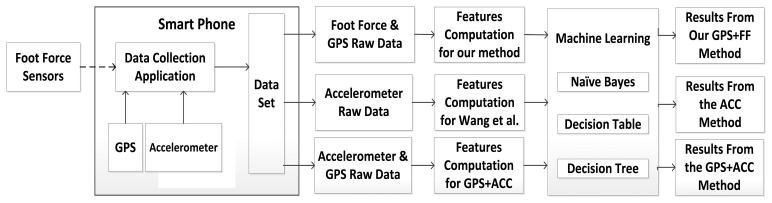
Architecture of the mobility activity recognition system.

**Figure 2.**
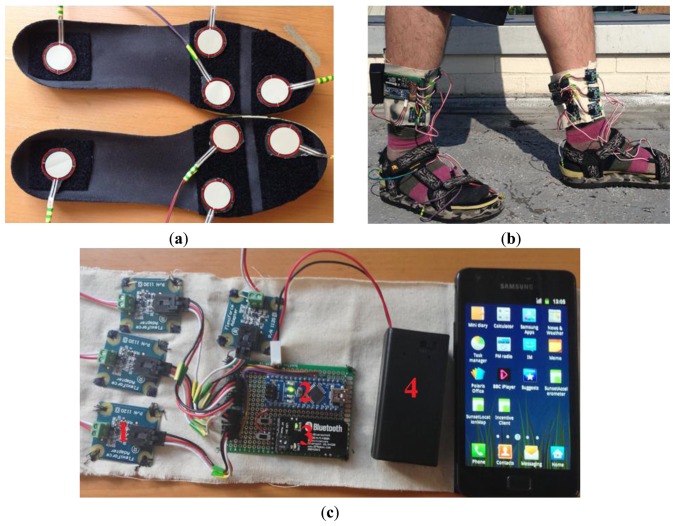
Experiment equipment: (**a**) two insoles with 8 Flexiforce sensors instrumented; (**b**) the wearable sensor prototype; (**c**) The foot force sensing system and a Samsung galaxy II smart phone.

In order to show the usefulness of the GPS + FF sensor-based method, the mobility activity recognition system as shown in Figure 1 is proposed. Thus, GPS + FF mobility activity recognition system also collects the data from different sensors simultaneously. Sensors include foot force sensors (as shown in Figure 2), mobile phone GPS and mobile phone accelerometer. For comparison purpose, in addition to the mobility activity recognition results from GPS + FF, the mobility activity recognition results from both an accelerometer-based method [20] and a GPS + ACC based method are also generated. With this system, a user only needs to perform required activities, once, to collect data for three different methods. This eliminates the variability caused by different data samples, which may affect the comparison results. Hence the evaluation results are better able to evaluate the GPS + FF method through comparing it with both an accelerometer-based method, e.g., [20], and a GPS+ACC based method as baselines.

There are three main data processing phases in the system: Raw Data Collection, Feature Computation and User Activity Recognition. In the raw data collection phase: The data from foot force sensors, GPS and accelerometer are collected simultaneously during different performed activities by the smart phone. The data is saved in CSV format. An Android application has been designed and implemented to enable volunteers to clearly label the data with the mobility activity to aid classification validation.

In the feature computation phase: the raw data collected from the previous phase is extracted without any prepossessing. This means all sensor error in daily life is presented to the feature computation phase, in order to meet the Sensor Error Tolerance requirement given in Section 3.1. Three sets of sensor data features are computed: ACC, GPS + ACC and GPS + FF. The former two methods are used as a baseline for comparison uses.

In the mobility activity recognition phase: the output from the data collection phase is converted as the input for the machine learning tool. Three different machine learning algorithms: naive Bayes, Decision Table and Decision Tree, are selected as computationally light-weight for use in mobile devices [56]. The outputs from this phase included the results for both human posture recognition and transportation mode recognition using three different methods: the GPS + FF method, ACC only method and a GPS+ACC based method for comparison.

### 3.4. Raw Data Collection

#### 3.4.1. Participants

All study procedures were approved by the Research Ethics Committee at Queen Mary University of London and participants signed a written informed consent form. Data collection took place over a 12-months period from December 2011 to December 2012. Each of the human postures and transportation modes (sitting, standing, walking, cycling, bus passenger, car passenger and car driver) were performed by 10 volunteers (six male; four female) with an age range from 24 to 56.

During data collection, volunteers had the liberty of carrying the mobile phone device in any orientation and position that they desired, such as near the waist, in a knee-high pocket, in a back-pack, in the top jacket and by hand. The data collected totalled 12,104 samples (each sample is 8s duration), of which 2,198 samples are from standing, 2,032 samples are from sitting, 1,584 samples are from walking, 1,603 samples are from cycling, 1,892 samples are from riding buses, 1,437 samples are from taking car/taxi and 1,358 samples are from driving (Section 3.4.2).

#### 3.4.2. Equipment

During the data collection procedures, each participant carried a Sumsung Galaxy II smart phone and wore a pair of special insoles. Each of the special insoles was instrumented with four Flexiforce sensors (eight sensors in total) as shown in Figure 2a. The sensitive range of each Flexiforce sensor is from 0 kg to 12 kg with linearity error less than ±3%. The response time is less than 5 microseconds. Both insoles are instrumented with force sensors in order to monitor the ground reaction force shifting between left foot and right foot. The sum values of the four sensors readings form the force readings of one foot. It has been shown that four force sensors arranged under the supporting bones of the foot and mounted inside the shoe can obtain accurate ground reaction force value [13]. Hence, four Flexiforce sensors have been mounted directly under the major weight-bearing points of each foot in order to cover the force reaction area of heel, forefoot, and toe for both feet as shown in Figure 2a. The reason for choosing both heel and forefoot as the focused area is based on a previous work, which has proved the usefulness of measuring force reaction in these (two) underfoot placements [13,17,42]. The distribution of sensors is based on the distribution of ground reaction force of each foot during walking. The distribution of ground reaction force on in-shoe plantar pressure during walking is illustrated in [57]. For each foot, the force peaks are mainly generated from one point at the heel and three points at the forefoot.

All Flexiforce sensors are interfaced to the smart phone *via* a Bluetooth connection from two designed foot force sensing systems (as shown in Figure 2b). The foot force sensing system (as shown in Figure 2c) is implemented using four adaptors (http://www.phidgets.com/docs/1120_User_Guide) (marked as 1 on Figure 2c), one Arduino Nano Board (http://arduino.cc/en/Main/ArduinoBoardNano) (marked as 2 on Figure 2c), one Bluetooth module (marked as 3 on Figure 2c), and one 9 V battery box (marked as 4 on Figure 2c).

All Flexiforce sensors are interfaced to the smart phone wirelessly. Flexiforce sensor readings are set to 35 Hz. The mobile phone embedded GPS is set to 1 Hz over the Android 2.3.3 OS platform. The smart phone embedded accelerometer (it is an in-built 3-D accelerometer, whose sensitivity is programmed from −2 g to + 2 g (g = 9.8)) (for comparison purpose) is set to 35 Hz according to the settings used in [20]. All raw sensor data from Flexiforce force sensors, mobile phone embedded accelerometer and mobile phone GPS were collected simultaneously during each activity (Section 3.3).

### 3.5. Feature Extraction

A uniform-duration (8 seconds window) sample (without overlap) as used in [20] is used by all three methods. For the collected sensor data, no noise filtering is carried out. This means all the sensor errors arising via daily living environment was presented to the feature computation phase.

For the ACC method, the following 11 features (as described in [20]) are extracted from the accelerometer data: mean, standard deviation, mean crossing rate, third quartile, sum and standard deviation of frequency components between 0∼2 HZ, ratio of frequency components between 0∼2 HZ to all frequency components, sum and standard deviation of frequency components between 2∼4 HZ, ratio of frequency components between 2∼4 HZ to all frequency components and spectrum peak position.

For the comparative use of the GPS + ACC method, the following 14 features are extracted from each window segmentation of data collected from both GPS speed and magnitude series of the accelerometer data: the mean, maximum and standard deviation of the GPS speed; mean, standard deviation, mean crossing rate, third quartile, sum and standard deviation of frequency components between 0∼2 HZ, ratio of frequency components between 0∼2 HZ to all frequency components, sum and standard deviation of frequency components between 2∼4 HZ, ratio of frequency components between 2∼4 HZ to all frequency components and spectrum peak position.

Then for the GPS + FF method, the following seven time-domain features are extracted from each window segmentation of data collected from both GPS and foot force sensors: the mean value, max value and standard deviation of the GPS speed; overall mean value, overall standard deviation and max value of foot force readings from both the left insole and the right insole; cross-correlation coefficient between the left foot force and the right foot force.

For each window for the foot force data, “Lx” is used to denote the force values from the left foot and “Rx” to denote the force values from the right foot. The mark “X” represents the number of the sampled value. For a data window with N samples (N is the window size), the following set of value pairs is generated (L_1_, R_1_), (L_2_, R_2_), …, (L_N_, R_N_).

The overall mean value of force readings from both feet can determine whether or not the whole body weight is supported by the user (e.g., when sitting, part of a user weight is supported by the chair). The overall mean value “MA” of the ground reaction force from both insoles is generated is as follows:

(1) $$M_{A}=\bar{L}+\bar{R}=\frac{\sum_{i=1}^{N}L_{i}}{N}+\frac{\sum_{i=1}^{N}R_{i}}{N}$$

In the equation above, L̄ and R̄ are the mean force values from both the left foot and right foot.

The overall standard deviation “SA” of the foot force generated is calculated using the following equation:

(2) $$S_{A}=\frac{S_{L}+S_{R}}{2}=\frac{\sqrt{\sum_{i=1}^{N}{\left(L_{i}-\bar{L}\right)}^{2}}+\sqrt{\sum_{i=1}^{N}{\left(R_{i}-\bar{R}\right)}^{2}}}{2}$$

In this equation, S_L_ and S_R_ are the standard deviations of force readings from both left foot and right foot.

Besides the two features mentioned above, another key feature is the cross-correlation coefficient between left foot force and right foot force. This is used to monitor the regular pressure shift between both feet. The cross-correlation coefficient between the left foot force and the right foot force is useful in detecting periodical foot related activities that need both feet to generate force in turn, such as cycling and walking. The cross-correlation coefficient between the left foot force and the right foot force is computed from the following equation:

(3) $$\gamma_{LR}=\frac{\sum_{i=1}^{N}(L_{i}-\bar{L})(R_{i}-\bar{R})}{S_{L}S_{R}}=\frac{\sum_{i=1}^{N}(L_{i}-\bar{L})(R_{i}-\bar{R})}{\sqrt{\sum_{i=1}^{N}{\left(L_{i}-\bar{L}\right)}^{2}\sum_{i=1}^{N}{\left(R_{i}-\bar{R}\right)}^{2}}}$$

In the equation above, γ_LR_ is the correlation coefficient between the left foot and the right foot force patterns. The range of γ_LR_ is between −1 and 1. In a positive relationship as the left foot force increases, the right foot force tends to increase too. The value will be 1. In a negative relationship as the left foot force increases, the right foot force tends to decrease. The value will be −1. If the left foot force and right foot force are independent, then the coefficient will tend to be zero, e.g., this value tends to be zero, when a user is sitting.

### 3.6. Mobility Activity Recognition

Three light-weight classifiers, naive Bayes (NB), Decision Tree (DTr) J48 and Decision Table (DTa) provided by WEKA toolkit are used to compare the performance of these three different (ACC, GPS + ACC, GPS + FF) methods (see Figure 1) [56].

For the ACC method, all features computed from accelerometer readings (see Section 3.5) are fed into the above three classifiers to generate the results for both human posture and transportation mode recognition.

For the GPS + ACC method, all features computed from both accelerometer and GPS readings (see Section 3.5) are fed into the above three classifiers to generate the results for both human posture and transportation mode recognition.

For the GPS + FF method, all features computed from foot force sensors readings (see Section 3.5) are fed into the above three classifiers to generate the results for human posture recognition, while all features computed from both foot force sensors and GPS readings (see Section 3.5) are fed into the above three classifiers to generate the results for transportation mode recognition.

All experiment data collected from 10 volunteers are equally divided into 10 folds. A 10-fold cross validation mechanism is used for evaluation, which includes data from each subject in both training and testing sets [58].

## 4. Results and Analysis

### 4.1. Experiment Objectives

The following Experiment hypotheses were devised in order to illustrate the benefits of the use of GPS + FF sensors to profile user mobility activities, *versus* typical methods based upon either ACC only or on GPS + ACC combinations:

1. FF sensor data clusters differently with respect to different human postures and human-powered (standing, sitting, walking and cycling) mobility compared to typical accelerometer sensor data (see Section 4.2).
2. GPS and FF sensor data clusters differently with respect to different (human-powered and motorised, e.g., walking, cycling, bus passenger, car passenger and car driver) transportation or mobility modes compared to typical accelerometer data (see Section 4.2).
3. The FF method for human posture and human-powered mobility recognition can outperform a typical ACC-based (ACC only) method for detecting these (see Section 4.3.1).
4. The GPS + FF method for transportation mode recognition can outperform both ACC-based method and GPS + ACC based method for detecting these (see Section 4.3.2).
5. The GPS + FF method requires less computational resources, in both the feature extraction phase and activity recognition phase than the ACC-based method and the GPS + ACC based method (see Section 4.4).

### 4.2. GPS, FF and ACC Sensor Data Clustering for Different Mobility Activities

**Figure 3.**
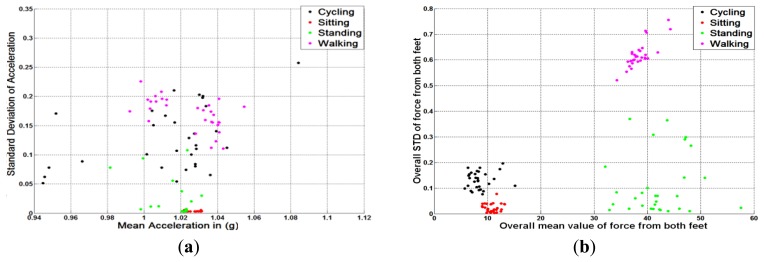
Clustering results of 120 samples from four human postures using (**a**) accelerometer *versus* using (**b**) foot force sensors.

**Figure 4.**
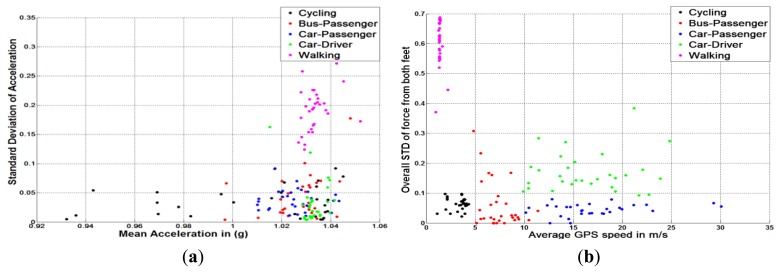
Clustering results of 120 samples from five transportation modes using (**a**) accelerometer *versus* using (**b**) the combination of foot force sensors and GPS.

One of the main design considerations Sections 1.2 and 3.1) is to minimise the computational load used for mobility activity classification. Hence, time-domain features, which require less computational resources than frequency-domain features [56], are selected. Figures 3 and 4 show the clusters of FF-based method and ACC-based method using only two basic time-domain features. Each different user mobility activity contains 30 different samples that were collected from 10 different subjects in daily living environment. These figures illustrate that if time-domain features are chosen, the GPS + FF method achieves better clustering than the typical ACC, and GPS + ACC methods. In addition, Figures 3 and 4 are actually preliminary results that lead to the main experiment hypotheses (Section 4.1).

Figure 3 illustrates the clustering results of different human postures using different methods. Samples from different postures are marked in different colours. Samples from cycling are in black, sitting are in red, standing are in green and walking are in pink. The left diagram in Figure 3 shows the clustering result of using an accelerometer. For each sample, the mean (X-axis) and the standard deviation (Y-axis) of the accelerometer readings are calculated according to Section 3.5. The right diagram in Figure 3 shows the clustering of measurements of different human postures in a similar manner to the left diagram, but using foot force sensors instead of accelerometer measurements.

It is noted that samples corresponding to sitting and standing are quite close to each other, with the lowest standard deviation values. This is because both postures exhibit quite similar acceleration patterns, which makes them hard to be differentiated using an accelerometer. Samples from both cycling and walking have a larger standard deviation compared to stationary postures. The diagram also shows a large overlap between walking samples and cycling samples. This is because the acceleration patterns from both walking and cycling activities sometimes are dominated by several other factors, *i.e.*, on-body placements, body motion, *etc.*, rather than by the activities themselves.

In contrast to accelerometer results, it is noted that mean values of FF measurements for sitting and standing are quite distinct (Figure 3). This is because the full user weight is sensed when standing, while only around a quarter of user weight is sensed when a user is sitting. Samples from both cycling and walking also differ. This is because both standard deviation and mean values of foot force readings from the walking samples are higher than those from the cycling samples (see Table 2).

Figure 4 shows the clustering results of different transportation modes using different methods. Samples from cycling are in black, bus-passengers are in red, car-passengers are in blue, car-drivers are in green and walking are in pink. The left diagram of Figure 4 shows the clustering results using accelerometer in terms of the mean value (X-axis) and standard deviation (Y-axis). It is noted that except for walking, measurement of the other transportation modes are similar. The reasons for this similarity are as follows. First, some transportation modes such as car-passenger and car-driver, the human movements are quite similar. Second, in many cases, the standard deviation values of acceleration from different transportation modes are dependent on multiple variables e.g., vehicle types, how the phone is being carried and the road conditions.

The right diagram of Figure 4 illustrates the clustering results of different transportation modes using foot force sensors and mobile phone GPS. For each sample, the average GPS speed (X-axis) and overall standard deviation of ground reaction force (Y-axis) of both feet (sensed during performing different transportation modes) have been calculated. This means each sample corresponds to one point in the two dimensional diagram as presented in Figure 4. From the right diagram of Figure 4, samples from walking, exhibit the highest foot force variance and the lowest average GPS speed, which are distinct from samples from other transportation modes. This is because the walking activity generates the most vigorous ground reaction force compared with other transportation modes. It is also found that samples from cycling, as another human powered transportation mode, have the second lowest average speed. With regard to different motorised transportation modes, bus-passengers have the lowest average GPS speed. This is because buses need to travel slower for safety consideration and stop regularly at bus stops. Although, samples from car-passengers and car-drivers have a very similar GPS speed, they are distinct in terms of variance of ground reaction force. This is because drivers need to step on both brake pedal and acceleration pedal.

### 4.3. Mobility Activity Recognition

For human posture recognition and transportation mode recognition, the overall accuracy from three selected classifiers has been presented. The detailed precision and recall results of each classifier are also given.

Accuracy tells us how well a method is able to identify positives and negatives correctly. Accuracy is defined as the sum of true positives and true negatives over the total number of classifications. Precision tells us how well a method is able to discriminate between true and false positives. Precision is calculated as the number of true positives over the total number of true positives and false positives. Recall tells us how well a method is able to recognize one particular mobility activity given all samples from this kind of mobility activity. Recall is calculated as the number of true positives over the sum of true positives and false negatives.

#### 4.3.1. Human Posture and Human Powered Mobility Recognition Using FF

**Figure 5.**
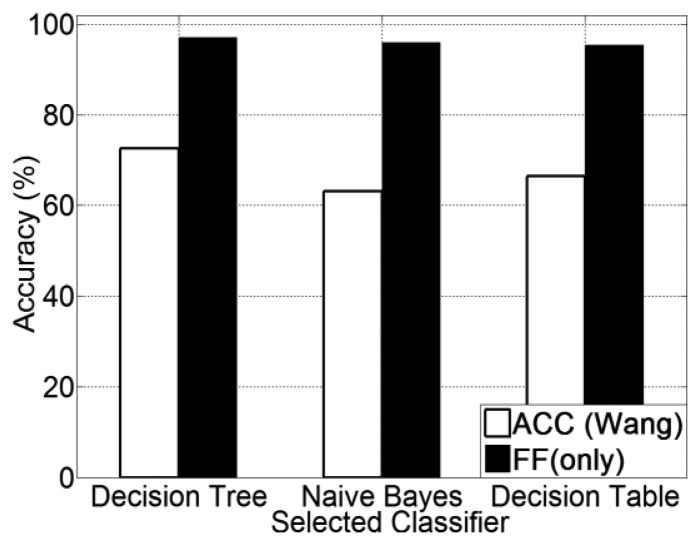
Human posture (and Human Powered Mobility) recognition results using different classifiers.

**Figure 6.**
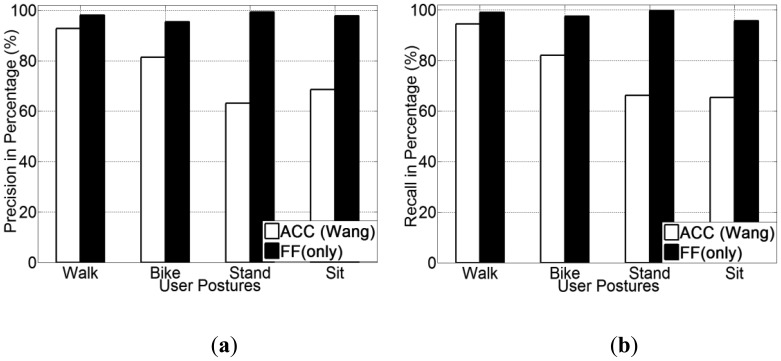
Human posture and mobility recognition results using decision tree: (**a**) precision; (**b**) recall.

**Figure 7.**
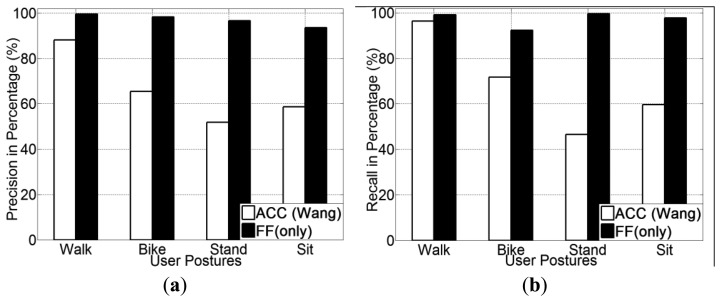
Human posture and mobility recognition results using naive Bayes: (**a**) precision; (**b**) recall.

**Figure 8.**
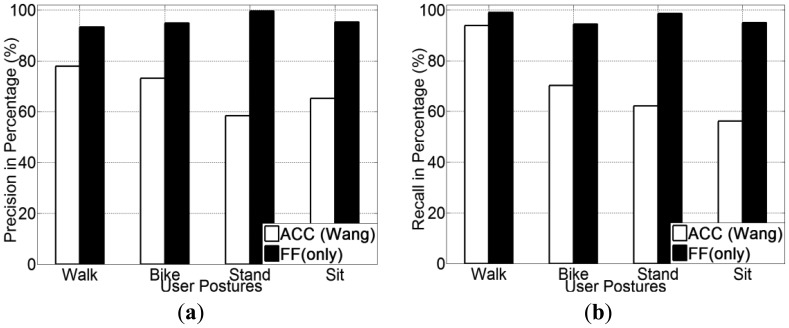
Human posture and mobility recognition results using decision table: (**a**) precision; (**b**) recall.

The experimental results for human posture recognition using ACC *versus* using FF (only) are presented in Figure 5. From Figure 5, it is noted that the foot force sensor method obtains a higher recognition accuracy than the accelerometer-based method, which was reproduced according to [20]. Among all three selected classifiers, the FF method achieves an accuracy of 96.1% on average, which is 28.8% higher than the accelerometer method (67.3% on average). In addition, the use of a decision tree (J48) classifier obtains the highest recognition accuracy for all three methods. The precision and recall for each human posture of each classifier are presented from Figure 6, 7 and Figure 8.

Regarding the precision and recall results, it is noted that the FF method outperforms the accelerometer based method in all aspects, especially in recognising cycling and in sub-differentiating the stationary postures into standing and sitting.

It is also noted that both methods perform equally well in detecting walking. This is a reasonable result, since there are three obvious stances in a normal human walking motion: heel strike, mid-stance and toe-off [12,59]. The accelerometer can detect the quite different acceleration patterns generated from these three stances, which are quite different compared with other human. Hence, the accelerometer-based method can detect walking posture at a high accuracy. The FF method can also detect foot force pattern variations generated from normal walking motion, the patterns of which are also unique in terms of both mean and variance.

From these figures, it is discovered that our method can detect the cycling at a higher accuracy (around 95%) compared with the accelerometer-based method (around 70%). This is because cycling also apparently differs from other types of human-powered mobility in terms of their foot force patterns. As people tend to power a bike by pedalling regularly when cycling, the foot force patterns generated are also distinct from other human postures (as shown in Table 2). While the accelerometer-based method, in many cases the acceleration patterns are mainly affected by the road conditions, rather than the posture itself. Based on this reason, in case of cycling over smooth roads, samples are quite similar with those from the stationary postures. On the other hand, for the case of cycling over rough roads, some samples are even similar to those from the walking posture. This variability introduces more false negatives.

**Figure 9.**
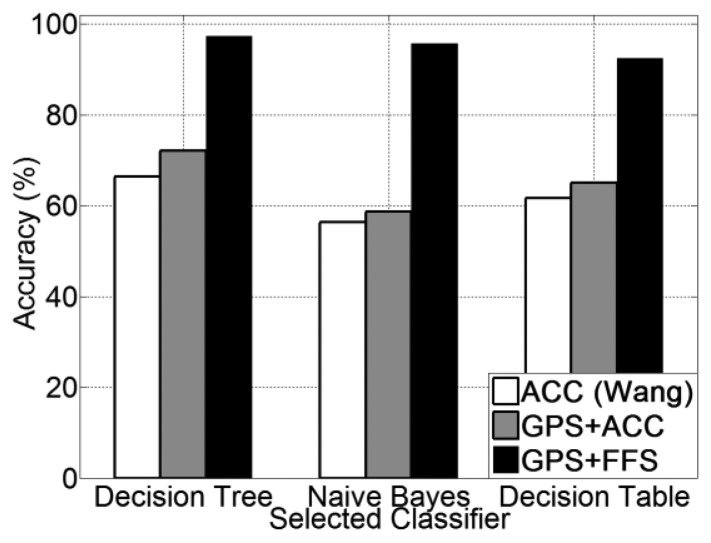
Comparison of some common features recognised by common 1st stage classifiers for human-powered and motorised transportation modes.

**Figure 10.**
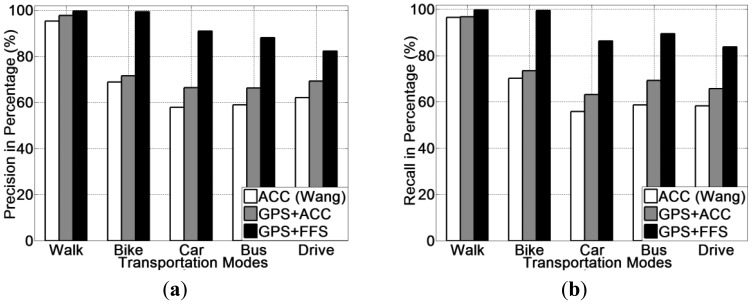
Transportation mode recognition results using decision tree: (**a**) precision; (**b**) recall.

For the case of recognising fine-grained human postures, it is remarked that the accelerometer-based method is unable to sub-differentiate stationary postures into sitting and standing. Both precision and recall for both sitting and standing postures are quite low, at a level of 50% (Figures 9 and 10). This is because the acceleration patterns from both postures are quite similar, even visually identical. Though the accelerometer-based method can differentiate human postures between stationary and non-stationary, it is not capable of sub-differentiating stationary posture (into standing and sitting).

However, our method in this case achieved an overall 95% accuracy on average in differentiating between sitting and standing postures. This is mainly because the amplitude of foot force patterns from both sitting and standing tend to be very different. In a standing posture, the whole user weight is fully supported by both feet, thus is sensed by the foot force sensors; while in a sitting postures, only part of user weight is supported by both feet. So for the case of standing, the amplitude of force sensed by the sensors from both feet is obviously higher than that of the sitting posture and unlike the accelerometer or GPS, FF can be used to recognise them.

#### 4.3.2. (Human-powered and Motorised) Transportation Mode Recognition using GPS + FF

Experimental results for all five transportation mode recognition using different methods (ACC, GPS + ACC and GPS + FF) are presented in Figure 9. From Figure 9, it is noted that the GPS + FF method obtains the highest recognition accuracy (95.1% on average). The second highest accuracy (65.9% on average) is achieved by the GPS + ACC method, which is around 5% higher than the accelerometer-based method [20] (61% on average). In addition, use of a decision tree (J48) classifier obtains the highest recognition accuracy for all three methods.

**Figure 11.**
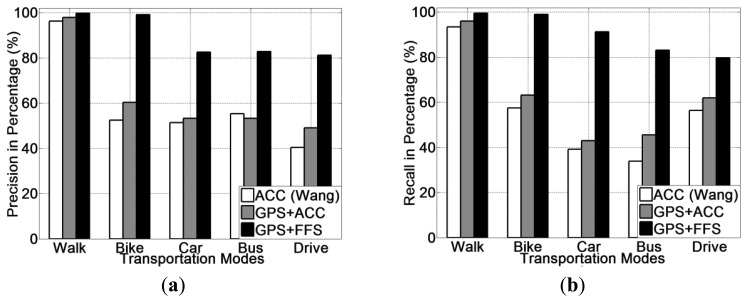
Transportation mode recognition results using naive Bayes: (**a**) precision; (**b**) recall.

**Figure 12.**
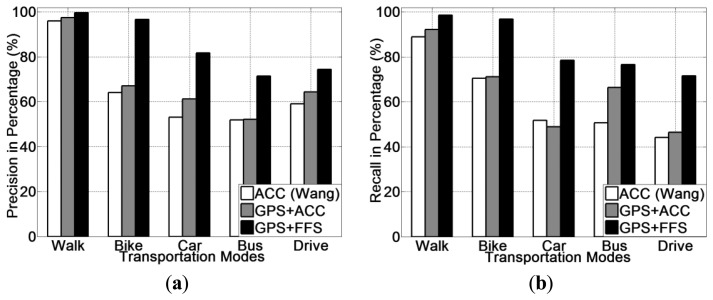
Transportation mode recognition results using decision table: (**a**) precision; (**b**) recall.

The precision and recall for each transportation mode of each classifier is presented from Figure 10, 11 and Figure 12. With respects to the precision and recall results, it must be remarked that the GPS + FF method outperforms the other two typical methods (accelerometer-based method and GPS-ACC based method) in all aspects, especially in recognising cycling and in sub-differentiating motorised transportation mode into car-passenger, bus-passenger and car-driver.

It is also noticed that all three methods perform equally well in detecting walking. There are three stances in a normal human walking motion: heel strike, mid-stance and toe-off [59]. Accelerometer can detect the acceleration generated from these three stances, which are quite different compared with other transportation modes in terms of variance. GPS can detect the travelling speed in real time (Table 2). Our method can also detect foot force patterns generated from normal walking motion, the variations of which are quite unique in terms of mean and variance.

From these figures, it is found that the accelerometer based method achieved the lowest accuracy in detecting cycling. This is because in many cases, the acceleration patterns that are mainly affected by the road conditions are similar with those instances from motorised transportation mode. This introduces a lot of errors from false negatives. With respect to the GPS + ACC based method, it is noticed that the accuracy for detecting cycling, increased but the improvement is little compared with the GPS + FF method. This is because there are still many motorised samples that exhibit similar characteristics in both acceleration and GPS speed with cycling. These are unable to be differentiated using the GPS + ACC method. It is also noted that the FF method can detect cycling at a very high accuracy (around 98%) compared with the two other methods (around 65%). This is because cycling differs from other transportation modes in terms of both mean GPS speed and foot force patterns. As Table 2 shows, the average speed of all samples from cycling is around 2.5 m/s. This is different from both walking (around 1.3 m/s) and motorised transportation modes (around 6.8 m/s). Besides, as people need to power the bike by pedalling regularly when cycling, the foot force patterns generated are also distinct from other transportation modes (as shown in Table 2).

For the case of sub-classification of motorised transportation mode, it is noted that the instances from one motorised mode are easily misclassified as those of another motorised mode (or even cycling) using either a typical accelerometer-based method or a GPS + ACC based method. Motorised modes were sometimes mistaken as cycling since sometimes a bike exhibits a similar speed and acceleration to a slower moving vehicle. Moreover, samples from car-driver and car-passenger are identical in terms of both the GPS speed and acceleration patterns. In most cases, the acceleration is affected by the vibration of the vehicle propulsion and that caused by road conditions. This makes motorised modes very hard to be differentiated by any classifiers for acceleration data.

The GPS + FF method in this case achieved an overall 95% accuracy on average. This is mainly because foot force patterns in different sub-motorised modes tend to be different. As in the driving cases, people need to step on both the acceleration and breaking pedal regularly in order to control the car. In the bus cases, people may stand and walk around inside the bus, which would almost never happen for a car passenger. Moreover, the GPS speed patterns from bus is also different with samples from private cars, since buses tend to stop more regularly at bus stops and to move slower than private cars, including taxis, for safety consideration.

With respect to results obtained from the GPS + FF method, it is noted that some instances of driving have been mistaken as being bus-passenger. This is because in some cases, when a user was moving around in a bus, the foot force patterns tend to be similar to stepping on pedals when driving. Some instances for driving have also been mistaken as being car-passenger. These errors occurred during slow speeds or after stopping for a period of time. In these cases, foot force patterns tend to be similar, since drivers tend to be stationary and were not operating on the pedals.

To conclude, the results above show that the GPS + FF method recognised both human posture and transportation mode at the same time. The GPS + FF method achieved the overall recognition accuracy at a level of 90%, especially in detecting cycling and sub-classifying motorised transportation mode. The GPS + FF method also achieved a more fine-grained mobility activity recognition capability, in terms of sub-differentiating stationary postures into standing and sitting and sub-differentiating motorised transportation mode into bus-passenger, car-passenger and car-driving. Hence, these results also show that the GPS + FF system meet both “Wider and Fine-Grained Range Mobility Recognition Capability” and “High Mobility Classification Accuracy” requirements as depicted in Section 3.1.

Moreover, during the data collection, as all participants had the liberty of carrying the mobile phone device in any orientation and position desired, hence the “No On-Body Placement Restrictions for accompanied mobile devices” requirement (as depicted in Section 3.1) has been met. Besides, as there is no data prepossessing, which means all sensor data errors were present in the training data for the chosen classifiers, the “Sensor Error Tolerance” requirement (as depicted in Section 3.1) has been met.

### 4.4. Computational Complexity

As depicted in Section 3.1, computational-load is an important concern for mobile phone sensing applications, because the smart phone has limited resources and supports a range of tasks including higher priority communication. Most of the surveyed work is based upon an analysis of frequency domain features, which are quite computationally expensive to perform on the mobile device [44].

The computational complexity of user activity recognition systems mainly resides in two main aspects: feature computation phase and transportation mode classification phase. In the feature computation aspects, the GPS + FF method tend to consume less computational resources as only several basic time-domain features (included mean, standard deviation and max) are required. In contrast, the typical accelerometer-based methods normally derive many frequency-domain features (frequency components between 0∼2 HZ, spectrum peak position, *etc.*). Since all raw data collection is in the time-domain and because the frequency domain features require Fourier Transforms, these impose higher computational loads on mobile devices [56].

With respect to the mobility activities classification, the computational load for a classifier depends on the complexity of the trained model [56]. As Table 3 shows, given the same set of training samples, the classifiers trained by GPS + FF method have a reduced complexity compared with the same classifiers trained by both the accelerometer-based method and the GPS + ACC based method. With regard to the decision table classifier, The FF method only requires 1/6 of the rules that is required by Wang's method. On the other hand, the size of decision tree classifier trained by the GPS + FF method is also much smaller than that trained by the other two methods. For example, the size and No. of leaves trained by the GPS + FF method are 47 and 24, while size of No. of leaves trained by the accelerometer are 1,377 and 689, by GPS + ACC are 1,901 and 951. To conclude, compared with both an ACC only method and a GPS + ACC based method, the GPS + FF method saves computation in both feature computation phase and the final classification phase. Hence, the “Lightweight Local Mobility Data Computation” hypothesis as depicted in section 3.1 has also been met.

The hyperparamters of the decision tree and decision table classifiers are based upon the default settings in the WEKA tool. The size of the tree, or the number of rules may vary with different hyperparameter settings. As long as the same hyperparameter setting are applied uniformly across the classifiers, the discrepancy between sensor-based methods should remain similar even when different hyperparameter settings are used for the classifiers [60]. As the main purpose of the paper is to validate the value add of the new GPS+FF method, the optimum hyperparameter configuration will be considered as further work.

### 4.5. User Variation

With respect to the “Reduced Training to Classify Individuals” requirement (Section 3.1) another factor that affects the usability and feasibility of the mobility activity recognition system is whether or not the system would work for new users without much individual user-specific training. To assess this, two distinct experiments are performed: firstly, a 10-fold cross validation, where the classifier is trained with all users; secondly, leave-one-user-out mode, where classifiers are trained with all but one user (nine out of ten) and tested with the one not in the training set. The results for the 10-fold cross validation have already been presented and analysed in Sections 4.2 and 4.3.

Table 4 shows the results from the leave-one-user-out test on GPS + FF method. When training and testing is done on an individual user basis, the overall accuracy decreases by 1.4% compared to a generalised classifier that is trained and tested on all users. Thus, creating user specific classifiers, decreases the accuracy, although the loss in accuracy is minimal when compared with generalised classifiers.

With the leave-one-user-out mode, the GPS + FF new method achieved an average accuracy of 93.6% and a minimum accuracy of 87.9% is obtained as Table 4 shows. Based on the results, one can conclude that certain users might be unique and that a training set is necessary (that has a broad range of how activities could be performed). Different users may perform mobility activities differently, *i.e.*, different people have different walking, cycling, and driving styles. Some people may tend to use the forefoot more compared with others who use their heal more. This does not affect the user mobility accuracy because the overall ground reaction force from each foot is sensed, *i.e.*, the user variation differences are marginal compared with the difference in features used for detecting walking. However, some differences from other (non-walking) activities may affect a specific user. For the users that had the worst performance in terms of accuracy (user 2, user 3 and user 9), the decrease in performance mainly came in the cycling, bus-passenger and car-driver for which individuals often have different styles both in terms of foot force frequency and GPS speed intensity. For example, a user may cycle intensively, which generates quite different patterns (for both GPS speed and FF) compared with others who cycle more moderately. People also have different habits when taking a bus, e.g., some people like to be seated, some people prefer standing, or leaning against a bus, inside. These differences mean FF patterns may vary when detecting the bus-passenger mode. Driving styles also differ from user to user, e.g., some users tend to drive more intensively than other users. Though the different styles do affect the accuracy in detecting a specific user, the accuracy is still relatively high, at a level of 85%. It is also observed that the accuracy for a new user can be increased with a broader range of training data that includes samples of these variations.

The results from both experiments indicate that it is possible to achieve good performance without requiring users to provide specific training data as long as the training set contains enough variation in terms of each user activity. In this study, foot force normalisation is used to eliminate the discrepancy in terms of user weight. All user foot force values are normalised by taking the user weight as one unit (e.g., a 50lb foot force reading is normalised as value of 0.5 given the user weight is 100 lb). It is also found that users with different weights tend to have similar foot force patterns for one type of activity after normalisation. As Table 4 shows, for all ten participants in these experiments, the foot force values after normalisation are around 65% for walking and 18% for cycling, 53% for being bus-passenger, 21% for being car-passenger and 35% for driving. Even with 10 individuals, the minimum accuracy level was still above 87%. Compared to this, the ACC method only achieved a mean accuracy of 55.8% and a 49.3% minimum accuracy, whereas GPS + ACC achieved a slightly better accuracy with a 63.4% mean and a 57.2% minimum accuracy. These results illustrate that accelerometer-based methods require more user specific training than the GPS-FF-based method does.

## 5. Discussion

In this new GPS + FF mobility activity recognition system, the GPS sensor is only used to measure the velocity. It can be replaced or combined with other transceiver type position determination sensors, e.g., GSM, WiFi, for speed detection. The reasons why transceiver type positions sensors are chosen for speed detection rather than inertial sensors, e.g. tri-axial accelerometer, are as follows. First, speed detection involves temporal aggregation of acceleration readings in a mobile phone and this is not accurate, especially under daily use circumstances. This is mainly because there is no fixed placement of how users carry their mobile phones. These frequent changes of the phone's position and orientation may introduce large errors. In addition, the error in using temporal acceleration aggregation for speed detection propagates dramatically as the distance increases. Secondly, tri-axial accelerometer based speed detection is not able to provide other valuable information, e.g., user locations, during daily activities. Some combination of the user spatial context, location and other GIS information can be used to further improve the mobility detection in future work. For example, through knowing that a user is travelling by bus, and by matching user location sequences with a specific bus routes, one can infer that a user is travelling on a specific bus.

In this study, only the combined value of four FF sensors (instrumented for each insole) is monitored for each foot. The reasons why the separate values of the four different sensors in each foot are not considered in this work are as follows. First, as discussed in the introduction, multi-sensor based methods often require more resources, higher computation, and are normally harder to maintain. Second, the combined value the four FF sensors, properly distributed under the supporting foot bones, can better reflect the overall ground reaction force generated under each foot. In this way, we can better compute the cross-correlation coefficient between the left and right foot force as depicted in Equation (3). Third, by knowing the overall ground reaction force values generated under both feet, we can better normalise different user's foot force value variations by considering the user's weight as a whole. Finally, this work mainly focuses on assessing how well a combination of mobile phone GPS and wearable foot force (FF) sensors (GPS + FF) to recognise common daily mobility activities. More specific research regarding the usefulness of each sensor will be included in future work.

The GPS + ACC method, which has been used for comparison, is reproduced by extending the work of [22] in order to add GPS as an assistive sensor. Though a fair comparison can be achieved by using exactly the same GPS speed related features in both the GPS + FF method and the GPS + ACC method, an alternative method (with different GPS parameters, set of features, classification models, or hyperparameter settings) may lead to different results. More specific research regarding this will be included in our future work.

In order to be used in potential applications (Section 1) in daily life, in practice, the GPS + FF method has to be built as a commercial product. Here are some initial thoughts. Existing single shoe pedometer type footwear designs, e.g., Nike + iPod, could be advanced or modified to use FF sensors on both feet. Existing research prototypes have already used multiple FF sensors integrated into an insole, e.g., 16 sensors have been integrated in an insole [14]. In contrast, our GPS + FF method, which uses only four sensors per insole, is much cheaper in order to be commercialised. The source of power for the integrated FF sensors is a major issue. However, new material technologies, piezoelectric material may be used as a power generator to generate electricity during foot movement, such that in the near future, FF sensors can be powered by the insole during the impact of the foot while walking.

## 6. Conclusions and Outlook

In this work, the potential benefits of using mobile phone GPS in combination with a set foot force sensors to improve daily mobility activity recognition have been examined for the first time. Two normal stationary human postures (sitting and standing) and five daily transportation modes, including walking, cycling, bus passenger, car passenger and car driver, have been performed by ten different users. Postures and transport modes have been profiled and evaluated, by comparing the GPS + FF method with both an accelerometer-based method as in [20] and a GPS + ACC based method.

Given the sample size of this pilot and based on the classification algorithms employed, the new GPS + FF method has improved the user mobility activity recognition accuracy from 65% to 95%, on average. Our method achieves a wider range recognition capability which is capable of recognising both human posture and transportation mode simultaneously. Another key contribution of our work is to provide a more fine-grained mobility activity recognition capability in terms of both sub-differentiating stationary postures (into sitting and standing) and sub-differentiating motorised modes, *i.e.*, into bus passenger, car passenger and car driver with an accuracy of 92.8% on average. In addition, our method also has other advantages in terms of requiring less computational resources and requiring less individual training.

The reasons for a higher accuracy being achieved by the GPS + FF method are as follows. First, by making use of the foot force sensors, human-powered activities, such as walking and cycling, can be more easily differentiated using foot force patterns. Second, during driving, frequent steps on the accelerator and the brake pedal generate distinguishable foot force patterns. Third, bus passengers may stand and walk at least to get on and off the bus. This doesn't happen for car passengers and car drivers.

Another merit of the GPS + FF method is that it can also recognize human posture at the same time with recognizing transportation mode. This can contribute more in terms of better user context profiling for smarter services, e.g., to highlight information more for a decreased locus of focus when users are not seated.

Even, although a substantial accuracy improvement has already been achieved, the recognition accuracy for car-passenger, bus-passenger and car-driver is still relative lower compared with detecting walking and cycling. As our method can determine the transition points during daily travelling, the accuracy for transportation mode recognition could be further improved in combination with publicly available transportation information such as bus stop coordinates. For example, the transition point between walking and taking a bus should be near a bus stop, so the distance between this transition point and the nearest bus stop could be very useful in differentiating bus-passenger and (private) car-passenger. It is also noted that people often wait at a bus stop in order to take a bus.

In a practical system, one must consider energy efficiency. GPS could be switched from active mode to another mode depending on the values of the FF and or ACC sensors, regularly. All human powered activities can be determined by using FF (only) with a relatively high accuracy (98% for walking and 95% for cycling, see Section 4.3.1). When detecting these human powered activities, the GPS could be switched off to save energy, without significantly affecting the accuracy and then be switched on again when activity shifts are detected. For other motorised transportation modes, once the transportation mode is determined, the GPS can be switched off automatically and start again when the user starts walking, which indicates the user is transiting to another transportation mode [61]. In addition, foot force sensors can be powered by a portable battery (e.g., button battery). The data will be transmitted to and stored at the smart phone via a PAN. We leave exploring this energy efficiency extension as future work for this study.

## Figures and Tables

**Table 1.** Classification of related work concerning user activity recognition.

**Ref.**	**Sensor No.&Type**	**Sensor Placement**	**Mobility Activity**	**Features Extracted**	**Classifiers**	**Acc.**
[32]	5, (2-axis) ACC	Ankle, wrist, waist	Still, walk, Cycle, Run	Mean, energy, freq. domain entropy, correlation features, sum of the squared discrete FFT component, FFT DC component	NB, Decision Table (DTa), Decision Tree (DTr), Instance-based Learning	84%
[35]	ACC	Chest, trousers, jacket	Still, Walk, Run	Raw 3-axis vector readings from the Accelerometer	K-Nearest Neighbours (k-NN)	60%
[19]	ACC	Hip	Still, Walk, Run, Stairs	Mean, std. dev., Energy, Correlation	DTa; DTr (C4.5), k-NN, Support Vector Machines (SVM), naïve Bayes	84%
[49]	ACC, GPS, Audio	Trousers, hip, chest	Still, Walk, Run	Mean, std. dev., No. of accelerometer reading peaks; mean and std. dev. of DFT power of audio sensor readings	DTr (J48)	78%
[14]	32, FF	Under foot	Walk, Run, Stairs	6 force parameters, chronological incidence of occurrence, heel & toe vertical ground reaction. Sum of vertical ground reaction forces.	Artificial Neural Network (ANN), Hidden Markov Model (HMM)	93%
[22]	3, micro-phones 2, ACC	Wrist, Waist, shoulder, chest	Still, hammering, sanding	No. of peaks, mean amplitude of 2 ACCs, FFT coefficients	HMM	67%
[23]	ACC & GPS	Waist, chest, hand, In-bag	Still, Walk, Bike, Motorised	filters, sum of FFT coefficients from magnitude of the accelerometer; average GPS speed	Bayes Net, DTr (J48), SVM and HMM	89%
[21]	ACC & GPS	Right hip	Walk, run, bike, skate, Motorised	Mean, median & interquartile range for accelerometer, counts & steps and GPS mean speed	Discriminant function analysis (SAS PROC DISCRIM)	86%
[20]	ACC	Free	Still, Walk, Bike, Bus, Car	Mean, std. dev., mean-crossing rate, third-quartile, sum & std. dev. of frequencies 0∼4 HZ, ratio of frequency components (0∼4 Hz) to all components, spectrum peak position.	DTr (J48), k-NN, SVM	62%
[50]	GPS	Hand	Stop, walk, bike, car, bus	Mean, Max., std. dev. of velocity, Length	Bayes Net, DTr, Conditional Random Field, SVM	76%
[16]	GPS	Hand	Still, Walk, Motorised	Mean GPS speed, Temporal information (time of the day),	Hierarchical Conditional Random Fields	83%
[51]	GSM, Pedometer	Waist	Still, Walk, Motorised	Mean, Max, Variance of Euclidean Distance; correlation coefficient, No. of cell towers between 2 measurements	NB, SVM, AdaBoost and MultiBoost	85%

**Table 2.** Variations in average speed and foot force patterns in different transportation modes.

	**Walking**	**Cycling**	**Bus-Passenger**	**Car-Passenger**	**Driving**
GPS Speed (m/s)	1.3 ± 0.2	2.5 ± 1.2	5.2 ± 2.0	8.5 ± 5.2	7.8 ± 4.4
Left Foot Force (Percentage of one unit user weight)	67% ± 51%	18% ± 11%	53% ± 5%	21% ± 3%	35% ± 12%
Correlation coefficient between left & right foot force (chapter 3.5)	−0.47 ± 0.06	−0.33 ± 0.24	0.34 ± 0.42	0.01 ± 0.31	0.15 ± 0.27
Left Foot Force Pattern (5 min duration)	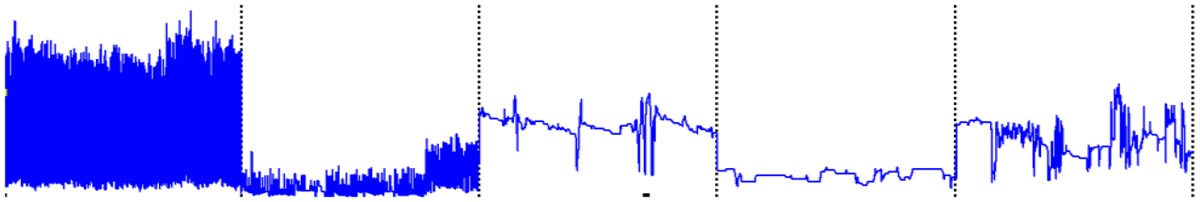				

**Table 3.** Number of tree leaves, tree size (number of nodes) and number of rules for classifiers.

	**Decision Table No. of Rules**	**Decision Tree**	

		**Size**	**No. of Leaves**
**ACC**	774	1,377	689
**GPS+ACC**	1,669	1,901	951
**GPS+FF**	123	47	24

**Table 4.** Decision Tree leave-one-user-out overall accuracy results.

User 1	94.6%	User 6	94.1%
User 2	87.9%	User 7	98.4%
User 3	93.1%	User 8	93.7%
User 4	96.1%	User 9	90.3%
User 5	95.3%	User 10	94.5%
		Average	93.8%

## References

[1] Poslad S. (2011). Ubiquitous Computing: Smart Devices, Environments and Interactions.

[2] Liu J., Goraczko M., Kansal A., Lymberopoulos D., Nath S., Priyantha B. Subjective Sensing: Intentional Awareness for Personalized Services.

[3] Consolvo S., Klasnja P., McDonald D.W., Avrahami D., Froehlich J., LeGrand L., Libby R., Mosher K., Landay J.A. Flowers or A Robot Army? Encouraging Awareness & Activity with Personal, Mobile Displays.

[4] Abie H., Balasingham I. Risk-Based Adaptive Security for Smart IoT in eHealth.

[5] Agapie E., Chen G., Houston D., Howard E., Kim J., Mun M., Mondschein A., Reddy S., Rosario R., Ryder J. Seeing Our Signals: Combining Location Traces and Web-Based Models for Personal Discovery.

[6] Froehlich J., Dillahunt T., Klasnja P., Mankoff J., Consolvo S., Harrison B., Landay J.A. UbiGreen: Investigating a Mobile Tool for Tracking and Supporting Green Transportation Habits.

[7] Mun M., Reddy S., Shilton K., Yau N., Burke J., Estrin D., Hansen M., Howard E., West R., Boda P. PEIR, the Personal Environmental Impact Report, as a Platform for Participatory Sensing Systems Research.

[8] Reddy S., Shilton K., Burke J., Estrin D., Hansen M., Srivastava M. (2009). Using context annotated mobility profiles to recruit data collectors in participatory sensing. Lect. Note. Comput. Sci..

[9] Streitz N., Nixon P. (2005). The disappearing computer. Commun. ACM.

[10] Dion J., Fouillot J., Leblanc A. (1981). Ambulatory Monitoring of Walking Using a Thin Capacitive Force Transducer.

[11] Hoyt R.W., Knapik J.J., Lanza J.F., Jones B.H., Staab J.S. (1994). Ambulatory foot contact monitor to estimate metabolic cost of human locomotion. J. Appl. Phys..

[12] Makihara Y., Rossa B.S., Yagi Y. Gait Recognition Using Images of Oriented Smooth Pseudo Motion.

[13] Lawrence T.L., Schmidt R.N. Wireless In-Shoe Force System (for Motor Prosthesis).

[14] Zhang K., Sun M., Kevin L.D., Xavier P.-S.F., Boozer C.N., Longman R.W. (2005). Assessment of human locomotion by using an insole measurement system and artificial neural networks. J. Biomech..

[15] Preece S.J., Goulermas J.Y., Kenney L.P.J., Howard D., Meijer K., Crompton R. (2009). Activity identification using body-mounted sensors–a review of classification techniques. Physiol. Meas..

[16] Liao L., Fox D., Kautz H. (2007). Extracting places and activities from gps traces using hierarchical conditional random fields. Int. J. Rob. Res..

[17] Tao W., Liu T., Zheng R., Feng H. (2012). Gait analysis using wearable sensors. Sensors.

[18] Saponas T.S., Lester J., Hartung C., Kohno T. (2006). Devices That Tell on you: The Nike+ ipod Sport Kit.

[19] Ravi N., Dandekar N., Mysore P., Littman M.L. (2005). Activity recognition from accelerometer data. AAAI.

[20] Wang S., Chen C., Ma J. Accelerometer Based Transportation Mode Recognition on Mobile Phones.

[21] Troped P., Oliveira N., Matthews C., Cromley E., Melly S., Craig B. (2008). Prediction of activity mode with global positioning system and accelerometer data. Med. Sci. Sport. Exer..

[22] Minnen D., Starner T., Ward J., Lukowicz P., Troster G. Recognizing and Discovering Human Actions from On-Body Sensor Data.

[23] Reddy S., Burke J., Estrin D., Hansen M., Srivastava M. Determining Transportation Mode on Mobile Phones.

[24] Subramanya A., Raj A., Bilmes J., Fox D. Recognizing Activities and Spatial Context Using Wearable Sensors.

[25] Gordon D. Group Activity Recognition Using Mobile Devices.

[26] Schutz Y., Herren R. (2000). Assessment of speed of human locomotion using a differential satellite global positioning system. Med. Sci. Sport. Exer..

[27] Witte T., Wilson A. (2004). Accuracy of non-differential GPS for the determination of speed over ground. J. Biomech..

[28] Accupedo C.L. Accupedo-Pedometer Widget. http://www.accupedo.com/.

[29] Apple Nike + iPod Sensor. http://www.apple.com/ipod/nike/.

[30] Lane N.D., Miluzzo E., Lu H., Peebles D., Choudhury T., Campbell A.T. (2010). A survey of mobile phone sensing. IEEE Commun. Mag..

[31] Mizell D. Using Gravity to Estimate Accelerometer Orientation.

[32] Bao L., Intille S. (2004). Activity recognition from user-annotated acceleration data. Perv. Comput..

[33] Parkka J., Cluitmans L., Ermes M. (2010). Personalization algorithm for real-time activity recognition using PDA, wireless motion bands, and binary decision tree. IEEE Trans. Inform. Technol. Biomed..

[34] Lee M.-W., Khan A.M., Kim T.-S. (2011). A single tri-axial accelerometer-based real-time personal life log system capable of human activity recognition and exercise information generation. Pers. Ubiquit. Comput..

[35] Brezmes T., Gorricho J.L., Cotrina J. (2009). Activity recognition from accelerometer data on a mobile phone. Lect. Note. Comput. Sci..

[36] Witten I.H., Frank E., Hall M.A. (2005). Data Mining: Practical Machine Learning Tools and Techniques.

[37] Lee B., Won-Chul B., Kim J.D.K., Chang Yeong K. (2011). Orientation estimation in mobile virtual environments with inertial sensors. IEEE Trans. Consum. Electron..

[38] Zhu C., Sheng W. (2011). Wearable sensor-based hand gesture and daily activity recognition for robot-assisted living. IEEE Trans. Syst. Man Cybern. Part A Sys. Hum..

[39] Yin J., Yang Q., Pan J.J. (2008). Sensor-based abnormal human-activity detection. IEEE Trans. Knowl. Data Eng..

[40] Baek J., Yun B.J. (2010). Posture monitoring system for context awareness in mobile computing. IEEE Trans. Instrum. Meas..

[41] Atzori L., Iera A., Morabito G. (2010). The internet of things: A survey. Comput. Netw..

[42] Veltink P.H., Liedtke C., Droog E., van der Kooij H. (2005). Ambulatory measurement of ground reaction forces. IEEE Trans. Neur. Syst. Rehab. Eng..

[43] Liu T., Inoue Y., Shibata K. (2010). A wearable ground reaction force sensor system and its application to the measurement of extrinsic gait variability. Sensors.

[44] Zhang X., Zhao Y., Duan Z., Liu Y. (2012). Design and test of a soft plantar force measurement system for gait detection. Sensors.

[45] Donati M., Vitiello N., De Rossi S.M.M., Lenzi T., Crea S., Persichetti A., Giovacchini F., Koopman B., Podobnik J., Munih M. (2013). A flexible sensor technology for the distributed measurement of interaction pressure. Sensors.

[46] Chon J., Cha H. (2011). Lifemap: A smartphone-based context provider for location-based services. IEEE Perv. Comput..

[47] Varkey J.P., Pompili D., Walls T.A. (2012). Human motion recognition using a wireless sensor-based wearable system. Pers. Ubiquit. Comput..

[48] Maekawa T., Kishino Y., Sakurai Y., Suyama T. (2012). Activity recognition with hand-worn magnetic sensors. Pers. Ubiquit. Comput..

[49] Miluzzo E., Lane N.D., Fodor K., Peterson R., Lu H., Musolesi M., Eisenman S.B., Zheng X., Campbell A.T. Sensing Meets Mobile Social Networks: The Design, Implementation and Evaluation of the Cenceme Application.

[50] Zheng Y., Liu L., Wang L., Xie X. Learning Transportation Mode from Raw Gps Data for Geographic Applications on the Web.

[51] Sohn T., Varshavsky A., LaMarca A., Chen M., Choudhury T., Smith I., Consolvo S., Hightower J., Griswold W., De Lara E. (2006). Mobility detection using everyday GSM traces. Ubiquit. Comut..

[52] Stenneth L., Wolfson O., Yu P.S., Xu B. Transportation Mode Detection Using Mobile Phones and GIS Information.

[53] Keally M., Zhou G., Xing G., Wu J., Pyles A. PBN: Towards Practical Activity Recognition Using Smartphone-Based Body Sensor Networks.

[54] Reddy S., Mun M., Burke J., Estrin D., Hansen M., Srivastava M. (2010). Using mobile phones to determine transportation modes. ACM Trans. Sens. Netw..

[55] Berchtold M., Budde M., Gordon D., Schmidtke H.R., Beigl M. Actiserv: Activity Recognition Service for Mobile Phones.

[56] Martín H., Bernardos A.M., Iglesias J., Casar J.R. (2012). Activity logging using lightweight classification techniques in mobile devices. Pers. Ubiquit. Comput..

[57] Chuckpaiwong B., Nunley J.A., Mall N.A., Queen R.M. (2008). The effect of foot type on in-shoe plantar pressure during walking and running. Gait Post..

[58] Kohavi R. (1995). A study of cross-validation and bootstrap for accuracy estimation and model selection. IJCAI.

[59] Yeh S., Chang K.H., Wu C.I., Chu H., Hsu J.Y. (2007). GETA sandals: A footstep location tracking system. Pers. Ubiquit. Comput..

[60] Thornton C., Hutter F., Hoos H.H., Leyton-Brown K. (2012). Auto-Weka: Automated Selection and Hyper-Parameter Optimization of Classification Algorithms.

[61] Zelun Z., Stefan P. A New Post Correction Algorithm (PoCoA) for Improved Transportation Mode Recognition.

